# Aspirin may take a back row seat for non-ST-segment elevation acute coronary syndrome after percutaneous coronary intervention

**DOI:** 10.1093/ehjcvp/pvaf090

**Published:** 2026-01-29

**Authors:** Patricia O Guimarães, Caio A M Tavares, Marcelo Franken, Pedro A Lemos

**Affiliations:** Academic Research Organization and Department of Cardiology, Hospital Israelita Albert Einstein, Av. Albert Einstein 627, São Paulo 05620-900, Brazil; Academic Research Organization and Department of Cardiology, Hospital Israelita Albert Einstein, Av. Albert Einstein 627, São Paulo 05620-900, Brazil; Instituto do Coracao (InCor), Hospital das Clinicas HCFMUSP, Faculdade de Medicina, Universidade de São Paulo, São Paulo 05403-000, Brazil; Academic Research Organization and Department of Cardiology, Hospital Israelita Albert Einstein, Av. Albert Einstein 627, São Paulo 05620-900, Brazil; Academic Research Organization and Department of Cardiology, Hospital Israelita Albert Einstein, Av. Albert Einstein 627, São Paulo 05620-900, Brazil

Reducing the time exposure to dual antiplatelet therapy (DAPT) after percutaneous coronary intervention (PCI) has been an alternative approach to avoid bleeding in patients with acute coronary syndrome (ACS).^1,2^ However, the population who may benefit more from P2Y12 inhibitor monotherapy without aspirin remains unclear. Several large trials testing aspirin-free regimens after PCI included a case-mix of acute and chronic patients, frequently excluded ST-segment elevation myocardial infarction (STEMI), and omitted aspirin only after 1 to 3 months of DAPT following PCI.^3,4^ The *Percutaneous Coronary Intervention Followed by Monotherapy Instead of Dual Antiplatelet Therapy in the Setting of Acute Coronary Syndromes* (NEO-MINDSET) trial, presented at the European Society of Cardiology Congress 2025, revealed that a strategy of very early aspirin withdrawal (median 2 days) should not be universally adopted since the non-inferiority of potent P2Y12 inhibitor monotherapy over DAPT for ischaemic events was not demonstrated (absolute risk difference of 1.47 percentage points with a 95% confidence interval [CI] −0.16 to 3.10, which was above the pre-specified margin of 2.5).^5^ NEO-MINDSET was a multi-centre, open-label randomized trial conducted in Brazil, including 3410 patients with STEMI or non-ST-segment elevation ACS (NSTE-ACS) who had undergone successful PCI with contemporary drug eluting stents within the first 4 days of hospitalization. As the protocol allowed for inclusion of all ACS spectrum, this provided an opportunity to assess treatment effects of early aspirin withdrawal according to ACS presentation. Overall, 62% of participants presented with STEMI and 38% with NSTE-ACS at baseline. This pre-specified secondary analysis, presented at the American Heart Association Scientific Sessions 2025, demonstrated a significant treatment effect heterogeneity for ischaemic outcomes: in STEMI, rates of death, myocardial infarction, stroke or urgent target-vessel revascularization were higher with monotherapy compared with DAPT (8.2% vs. 5.2%, hazard ratio [HR]: 1.60; 95% CI: 1.14–2.24), whereas in NSTE-ACS event rates were comparable between study groups (5.1% vs. 6.0%, respectively, HR: 0.84; 95% CI: 0.53–1.35; *P* for interaction = 0.030).^6^ The occurrence of major or clinically relevant non-major bleedings with monotherapy was less than half of what was seen with DAPT in both STEMI and NSTE-ACS. These data, although limited by the intrinsic restrictions of any subgroup analysis, raises an important question on whether aspirin is truly needed after NSTE-ACS for ischaemic protection or whether its antiplatelet effects are redundant on a background of potent P2Y12 inhibition. Indeed, STEMI and NSTE-ACS have different pathophysiology and patient profiles. In NEO-MINDSET, NSTE-ACS patients were older, had more cardiovascular risk factors and a larger burden of (multi-vessel) coronary artery disease compared with those with STEMI. Interestingly, while these characteristics may confer a higher long-term risk for recurrent atherosclerotic events in NSTE-ACS,^7^ in the acute setting, the relatively lower thrombotic milieu appeared to be adequately managed with potent P2Y12 inhibition. These findings are hypothesis generating and will help inform prospective trials testing monotherapy immediately after PCI to define whether aspirin still deserves a place at the table in this population.

## Data Availability

The data underlying this article were taken from the quoted references.
